# Genome-Wide Identification and Salt Stress-Responsive Expression Analysis of the *GmPLATZ* Gene Family in Soybean (*Glycine max* L.)

**DOI:** 10.3390/plants14132004

**Published:** 2025-06-30

**Authors:** Mingyu Wang, Zheyun Guan, Songquan Wu, Jingyong Zhang, Chunjing Lin, Yanyan Sun, Mingzhe Shen, Chunbao Zhang

**Affiliations:** 1 College of Agriculture, Yanbian University, Yanji 133002, China; mingyuwang1018@163.com (M.W.); arswsq@ybu.edu.cn (S.W.); 2 Key Laboratory of Hybrid Soybean Breeding of the Ministry of Agriculture and Rural Affairs/Soybean Research Institute, Jilin Academy of Agricultural Sciences (Northeast Agricultural Research Center of China), Changchun 130033, China; guanzy00@163.com (Z.G.); zhangjy@jaas.com.cn (J.Z.); lincj@jaas.com.cn (C.L.); sunyy@jaas.com.cn (Y.S.)

**Keywords:** soybean, *PLATZ* gene family, genome-wide analysis, expression profile, salt stress

## Abstract

The plant-specific *PLATZ* transcription factors play crucial roles in plant growth, development, and responses to abiotic stresses. However, despite their functional significance, *PLATZ* genes remain poorly characterized in soybeans. In this study, we conducted a genome-wide analysis of the *GmPLATZ* gene family and investigated their expression profiles under salt stress. We identified a total of 29 *GmPLATZ* genes in the soybean genome and systematically analyzed their physicochemical properties, conserved domains, evolutionary relationships, cis-acting elements, and expression regulation patterns. Subcellular localization predictions indicated nuclear localization for most *GmPLATZs*, except for *GmPLATZ5* and *GmPLATZ14*, which showed dual chloroplast–nuclear localization. A gene family expansion analysis indicated that 21 segmental duplication events were the primary driver of *GmPLATZ* diversification. A phylogenetic analysis classified the *GmPLATZ* genes into four subgroups, while gene structure and motif analyses revealed conserved zinc-binding domains and identified multiple cis-acting elements associated with light responsiveness, hormone signaling, and stress responses. Expression profiling showed tissue-specific expression patterns, with 13 *GmPLATZ* genes differentially expressed under salt stress, including root-preferential members (e.g., *GmPLATZ1*, *GmPLATZ10*) and leaf-preferential members (e.g., *GmPLATZ8*, *GmPLATZ9*). This study provides a theoretical basis for further investigation of *GmPLATZ* gene functions in soybean development and stress tolerance.

## 1. Introduction

Soybean is a pivotal food and oilseed crop, serving as an indispensable component of human life. However, in recent years, the expansion of saline–alkali soil has been driven by climatic and environmental changes [1]. As a salt-sensitive crop, soybean exhibits decreased emergence and germination rates under salt stress. Salinization disrupts the balance of K^+^ and Na^+^ in plants, leading to excessive reactive oxygen species (ROS) production and subsequent toxicity [2]. Excess ROS induce plasma membrane peroxidation, hinder photosynthesis, and trigger cell apoptosis, ultimately causing a 30% reduction in yield and a 23% decrease in 100-seed weight [3]. Given the profound impact of salt stress on soybean yield, extensive research on soybean salt tolerance has been progressively conducted. The SOS (Salt Overly Sensitive) pathway plays a crucial role in reducing cytosolic Na^+^ concentration in plants by promoting excessive Na^+^ efflux into the soil. The core components of the SOS pathway in Arabidopsis thaliana include *SOS1*, *SOS2*, and *SOS3*. *SOS1*, a plasma-membrane-localized Na^+^/H^+^ antiporter, facilitates Na^+^ extrusion from cells. The role of the Endosomal Sorting Complex Required for Transport (ESCRT) in salt tolerance has also been confirmed, as one of its components, VPS23A, enhances *SOS1* activity by promoting SOS2–SOS3 interaction and their plasma membrane localization [4]. Recently, it has been reported that Sorting Nexin 1 (SNX1) contributes to salt-induced plasma membrane localization and accumulation of *SOS1*. Mutants of the Arabidopsis transcription factors ARF7 and ARF19 are sensitive to salt stress, with reduced *SOS1* expression and elevated Na+ content observed in *arf7arf19* mutants under salt stress. Notably, ARF7 and ARF19 do not significantly bind to the *SOS1* promoter but activate *SOS1* transcription by binding to an AuxRE element within the first exon of the *SOS1*-coding region. Additionally, a RING-type E3 ubiquitin ligase, *CHYR1*, ubiquitinates and promotes the degradation of ARF7 and ARF19 proteins, thereby inhibiting *SOS1* expression and plant salt tolerance. Under high-salt conditions, *CHYR1* expression is suppressed, stabilizing ARF7 and ARF19 proteins, which ultimately enhances the *SOS1* expression and plant salt tolerance [5].

Transcription is regulated in large parts by transcription factors, which govern numerous biological processes, including development, signaling, and stress responses. Distinct transcription factor families, such as NF-Y, MYB, and WRKY, are critical for stress tolerance, whereas GRF regulates root, flower, and seed development, and AP2 participates in floral organogenesis and nodule formation [6]. Transcription factors contain one or more functional domains that activate or repress target gene expression by binding to their *cis*-acting regulatory regions. Combinatorial regulation further indicates that transcription factors cooperatively interact to orchestrate diverse cellular processes [7]. WRKY transcription factors (TFs) participate in the abscisic acid (ABA)-signaling pathway and function as cross-regulatory factors across diverse signaling networks. The overexpression of *GmWRKY12* [8] and *GmWRKY49* [9] has been shown to improve salt tolerance in transgenic soybean plants. bZIP transcription factors modulate soybean salt tolerance by promoting the expression of stress-responsive genes, with GmbZIP110 serving as a representative example [10]. The members of the NAC transcription factor family (NAM, ATAF1/2, and CUC1) share a conserved N-terminal DNA-binding domain. The overexpression of *GmNAC11* and *GmNAC20* may enhance the salt stress tolerance in soybean via the DREB/CBF-COR-signaling pathway [11]. Salt induction of *NAC1* (*GmSIN1*) promotes its binding to promoter regions, thereby upregulating 9-cis-epoxycarotenoid dioxygenase genes involved in ABA biosynthesis and respiratory burst oxidase homolog B genes (*GmRbohBs*) associated with ROS production in soybean. *GmSIN1*, *GmNCED3*, and *GmRbohBs* form a positive feedforward system, enabling rapid accumulation of ABA and ROS to effectively amplify the initial salt stress signals. The combined regulation of ABA and ROS levels enhances the salt tolerance in soybean [12]. When exposed to salt stress, the endogenous abscisic acid (ABA) level increases rapidly, activating sucrose non-fermenting 1-related protein kinases (*SnRK2s*). These kinases phosphorylate ABA-responsive element-binding protein/factor (AREB/ABF) transcription factors, regulating stomatal closure to cope with osmotic stress. Additionally, ABA maintains osmotic homeostasis by regulating the decomposition of starch into sugars and sugar-derived osmolytes. ABA acts in synergy with Ca^2+^ signaling to promote the Na^+^ efflux through the SOS3-SOS2-SOS1 pathway while simultaneously activating potassium channels to facilitate a K^+^ influx, thereby reducing the Na^+^/K^+^ ratio. The ABA activates the NADPH oxidase RBOHF, promoting the production of H_2_O_2_. As a signaling molecule, H_2_O_2_ further activates Ca^2+^ channels, and there exists complex signaling crosstalk among ABA, reactive oxygen species (ROS), and Ca^2+^ [13]. Consequently, a systematic investigation of transcription factors will unveil plant-specific mechanisms underlying secondary metabolism, phytohormone responses, and cell-type-specific trait development [14,15,16,17].

The plant-specific PLATZ (plant A/T-rich sequence and zinc-binding) transcription factor family comprises a novel class of zinc-dependent DNA-binding proteins that specifically recognize A/T-rich sequences in plants. Multiple sequence alignments reveal that PLATZ proteins possess two distinct zinc-binding regions essential for DNA binding, characterized by the conserved motifs C-x_2_-H-x_11_-C-x_2_-C-x_(4–5)_-C-x_2_-C-x_(3–7)_-H-x_2_-H and C-x_2_-C-x_(10–11)_-C-x_3_-C [18]. These DNA-binding proteins recognize and bind to *cis*-acting elements in promoter regions and modulate multiple biological processes, including DNA replication and transcriptional regulation [19]. Furthermore, their critical roles in plant defense mechanisms and acclimation responses across species have been extensively documented [20,21]. Since the initial discovery of *PLATZ1* in pea (*Pisum sativum* L.), the functional significance of PLATZ transcription factors in plant biology has garnered increasing scientific interest [18].

PLATZ transcription factors play critical roles in modulating growth and developmental processes across diverse plant species. In rice, the plant-specific PLATZ transcription factor GL6 interacts with C53 and C1 to positively regulate grain length by promoting cell proliferation in young panicles and grains [22]. SG6, which is expressed ubiquitously in cells but absent in the endosperm, enhances grain size and weight through its interaction with the DP transcription factor [23]. In maize (*Zea mays* L.), FL3 (*ZmPLATZ12*) encodes a PLATZ transcription factor that interacts with RPC53 and TFC1 to regulate endosperm development and storage reserve accumulation [24]. *ZmPLATZ2*, highly expressed in the endosperm, binds to the promoters of *ZmSSI*, *ZmISA1*, and *ZmISA2* to upregulate their expression, thereby driving starch synthase activity in maize endosperm [25]. PacBio iso-seq and RNA-seq analyses further demonstrate that PLATZ proteins mediate secondary growth in Populus stems [26]. A comparative transcriptome study of diploid cotton (*Gossypium hirsutum* L.) fibers from isogenic fuzzy-lintless (Fl) and normal fuzzy-linted (FL) lines revealed a downregulation of *PLATZ* in Fl lines at 10 days post-flowering compared to FL, highlighting its essential role in cotton fiber development [27]. Similarly, in sugarcane, differential expression of a *PLATZ* gene between mature and immature tissues of high-fiber genotypes suggests its involvement in transcriptional regulation of secondary cell wall biosynthesis [28]. In *Arabidopsis*, the PLATZ transcription factor ORESARA15 (ORE15) enhances leaf growth and delays senescence by modulating early cell proliferation dynamics and the GRF/GIF regulatory pathway [29]. Additionally, ABA-INDUCED EXPRESSION 1 (AIN1) regulates root elongation in response to ABA signaling [30]. Recent studies further indicate that ORE15 promotes root meristem enlargement by balancing the auxin–cytokinin signaling crosstalk [31].

Beyond their roles in plant growth and development, PLATZ transcription factors have been increasingly recognized as central regulators of abiotic stress responses. In *Arabidopsis*, both *AtPLATZ1* and *AtPLATZ2* have been reported to play positive regulatory roles in the seed desiccation tolerance (DT). Specifically, *AtPLATZ1* was confirmed through overexpression in a desiccation-intolerant mutant background to be crucial for seed DT [32], whereas *AtPLATZ2* acts as a transcriptional repressor that modulates the *Arabidopsis* salt-stress response by repressing the expression of *CBL4/SOS3* and *CBL10/SCaBP8* [33]. *PLATZ4* positively regulates plant drought tolerance and ABA sensitivity by binding to A/T-rich sequences in the promoter of *PIP2;8* [34]. Additionally, the *PhePLATZ1* is upregulated in response to drought treatments in moso bamboo. Heterologous overexpression of *PhePLATZ1* in *Arabidopsis* confers enhanced drought tolerance compared to wild-type plants [35]. Furthermore, drought stress significantly increases the expression of *PLATZ1* in maize, indicating that *PLATZ* genes play a crucial role in drought resistance [36].

Genome-wide characterization of *PLATZ* gene families has been investigated in multiple plant species, including rice (*Oryza sativa* L.) [22], maize [24], poplar (*Populus* L.) [26], cotton [27], sugarcane (*Saccharum* L.) [28], and *Arabidopsis* (*Arabidopsis thaliana* L.) [29]. Despite the global agricultural importance of soybean (*Glycine max* L.), comprehensive studies on its *PLATZ* gene family remains limited [37,38]. Nevertheless, various abiotic stresses, such as cold stress, salt stress, hormonal imbalances, and trace element toxicity, significantly impact the soybean productivity throughout its life cycle [39,40,41,42]. Thus, a genome-wide analysis of soybean *PLATZ* genes would therefore provide crucial insights into their functional mechanisms underlying stress responses.

## 2. Results

### 2.1. Identification and Analysis of the GmPLATZ Gene Family in Soybean

Multiple sequence alignment analyses identified 29 *PLATZ* genes in the soybean genome, which were designated as *GmPLATZ1* to *GmPLATZ29*, in line with their positions on the chromosomes. The lengths of GmPLATZ proteins ranged from 127 to 300 amino acids. The isoelectric points (pI) of these proteins fell within the range of 7.92–9.70, and their theoretical molecular weights (MWs) were between 14.72 and 36.72 kDa (Table S1). Specifically, *GmPLATZ13* exhibited the highest MW, whereas *GmPLATZ9* had the lowest MW. The theoretical isoelectric point (pI) values of all 29 proteins exceeded 7, which indicated that every protein in the soybean PLATZ family belonged to the category of basic proteins. The predicted subcellular localization of GmPLATZ proteins was predominantly nuclear, except for *GmPLATZ5* and *GmPLATZ14*, which showed dual localization in both chloroplasts and nuclei (Table S1). The 29 GmPLATZ proteins possess two distinct zinc-binding regions essential for DNA binding, characterized by the conserved motifs C-x_2_-H-x_11_-C-x_2_-C-x_(4–5)_-C-x_2_-C-x_(3–7)_-H-x_2_-H and C-x_2_-C-x_(10–11)_-C-x_3_-C (Figure S1) [18].

**Figure 1:**
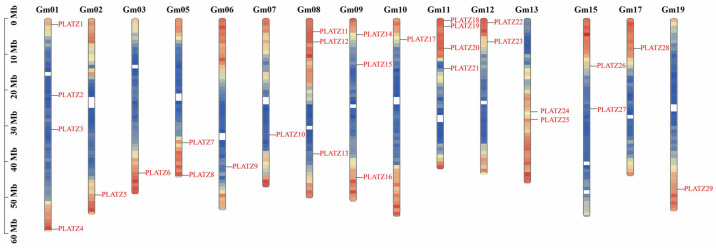
The distribution of *GmPLATZ* genes on soybean chromosomes. The vertical bars represent chromosomes.

The distribution of all the *GmPLATZ* genes across the fifteen chromosomes of the soybean is irregular. Four *GmPLATZ* genes were located on chromosome 1 and 11; followed by three genes on chromosome 8 and 9; two genes on chromosome 5, 12, 13, and 15; and one gene on chromosomes 2, 3, 6, 7, 10, 17, and 19. Through a chromosomal location analysis, we determined that chromosomes 4, 14, 16, 18, and 20 do not harbor any genes belonging to the *PLATZ* family (Figure 1).

### 2.2. Gene Structure and Motif Analysis of GmPLATZ

**Figure 2:**
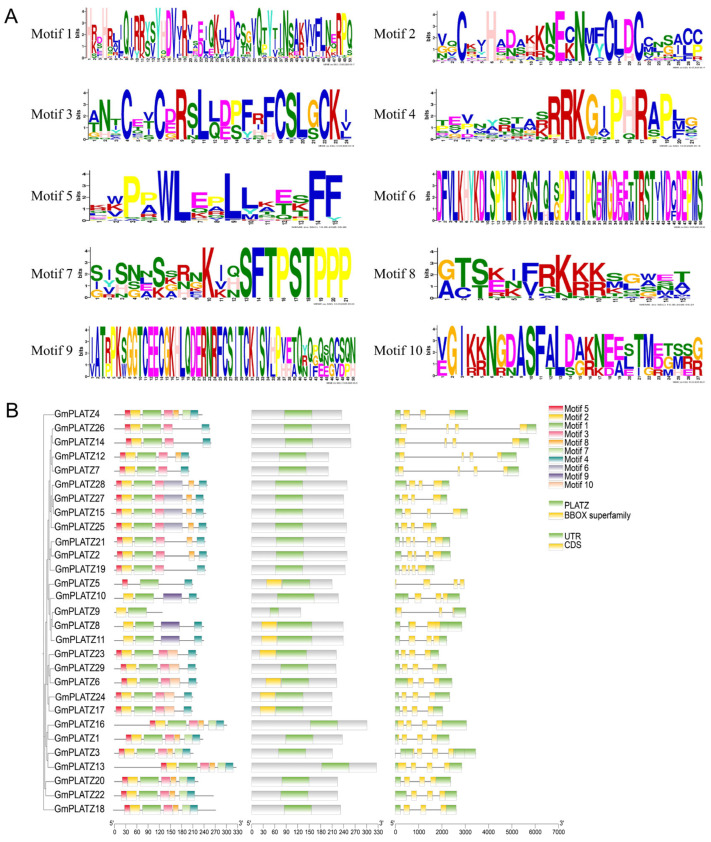
The gene structures, motif distribution, and conserved domains of GmPLATZs. (**A**) The motif logo of GmPLATZ proteins. (**B**) The motif distribution, conserved domains, and gene structure of the *GmPLATZ* family genes.

The differences in gene structure among family members are evident from the varying counts of introns and exons within the same gene family. To examine the structural features of *GmPLATZs*, the corresponding gene members were identified through a search of the soybean genomic DNA sequences. The exon–intron organization of *GmPLATZ* genes exhibited variations across subfamilies but remained consistent within each subfamily. As shown in Figure 2B, *GmPLATZ6* contains three exons and two introns, while other *GmPLATZ* genes typically possess four exons and three introns.

Amino acid sequences with high conservation may serve functions similar to those exhibiting a strong sequence similarity or uniformity. An analysis of the conserved motifs in GmPLATZ proteins revealed that members within the same subfamily shared highly comparable motif patterns. Using the MEME web tool, we detected 10 conserved motifs among GmPLATZ family members, with a subsequent analysis revealing significant conservation across these motifs (Figure 2A). All GmPLATZ proteins contain the conserved motifs 1 and 4, with motif 1 serving as the zinc-finger protein-binding site. Additionally, most members possess motifs 2, 3, and 5 (Figure 2B, Table S2). Notably, GmPLATZ8-11 form a distinct subfamily characterized by the absence of a second zinc-finger protein-binding site (Figure S1). These findings indicate evolutionary conservation among GmPLATZ proteins, potentially reflecting functional similarity.

### 2.3. Phylogenetic Tree Analysis of PLATZ Gene Family in Soybean

**Figure 3:**
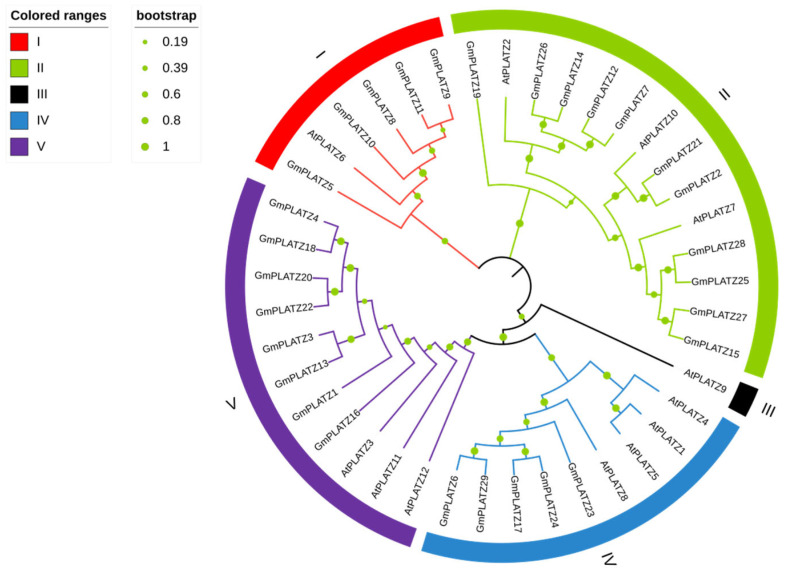
Phylogenetic analysis of PLATZ proteins in *Arabidopsis thaliana* and *Glycine max*. The sequences of proteins from *Arabidopsis thaliana* (At) and *Glycine max* (Gm) were analyzed and aligned using MEGA_X_10.1.7. The labels ranging from I to V signify the five distinct subfamilies identified within the tree.

To elucidate the evolutionary relationships of *GmPLATZ* genes, we constructed a phylogenetic tree comprising 41 *PLATZ* family members, including 12 from *Arabidopsis thaliana* (Table S3) and 29 from soybean. *AtPLATZ* genes can be classified into five subgroups, whereas *GmPLATZ* genes are grouped into four subgroups. Subfamily II is the largest in *GmPLATZs*, comprising 11 genes, while subfamily III exclusively contains *AtPLATZ9* and lacks any *GmPLATZ* genes (Figure 3). The phylogenetic tree suggests that GmPLATZs in the same group share conserved features, facilitating structure–function predictions in soybean based on the characterized model plant AtPLATZs. The Gm*PLATZ* family has a larger number of members compared to that in *Arabidopsis thaliana*. This difference might be attributed to multiple rounds of gene replication events that occurred during the evolutionary process.

**Figure 4:**
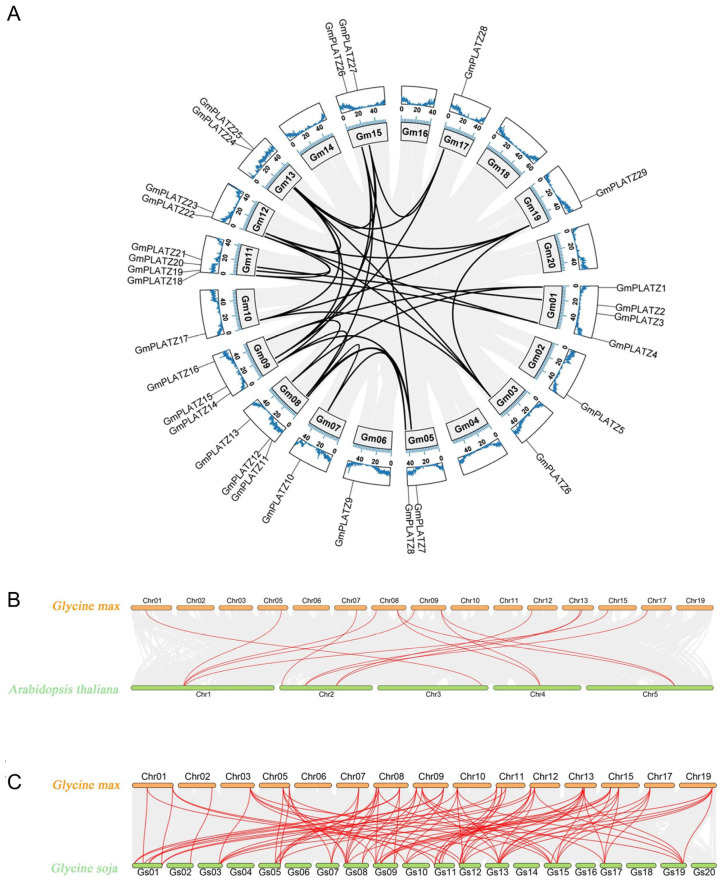
Evolutionary conservation of *GmPLATZ* gene organization across chromosomes. (**A**) The chromosomal distribution and synteny of *GmPLATZs* show (1) GC content, (2) soybean chromosomes, and (3) syntenic bloc. (**B**,**C**) The synteny of *PLATZ* genes was analyzed, respectively, for the combinations of *Glycine max*–*Arabidopsis thaliana* and *Glycine max*–*Glycine soja*. Specifically, red lines were applied to mark and make prominent the syntenic *PLATZ* gene pairs between soybean and the other species.

Gene duplication, which includes whole-genome duplication (WGD), tandem duplication, and segmental duplication, represents a fundamental evolutionary mechanism driving genomic diversification in plants. To investigate the expansion mechanism of the *GmPLATZ* gene family members in soybean, we performed an intra-genomic collinearity analysis. As shown in Figure 4A and Table S4, no tandem duplication events were detected among *GmPLATZ* genes. In contrast, 32 segmental duplication event pairs were identified. These results indicate that segmental duplication events, rather than tandem duplication, have been the predominant driver of the *GmPLATZ* family expansion. To further elucidate evolutionary relationships, we conducted a cross-species synteny analyses between soybean and two related species. A genome-wide comparison revealed 14 orthologous gene pairs between *Glycine max* (cultivated soybean) and *Arabidopsis thaliana* (Figure 4B, Table S5). Notably, this number increased substantially to 90 syntenic pairs when comparing *Glycine max* with its wild progenitor *Glycine soja* (Figure 4C, Table S6). This pronounced difference likely reflects genomic modifications occurring during soybean domestication and subsequent selection processes.

Next, we employed TBtools software (version 2.225) to calculate the Ka/Ks ratios for gene pairs. The Ka/Ks ratios of *Arabidopsis thaliana* and *Glycine max* were all less than 1 (Table S5), while for *Glycine max* and *Glycine soja*, only one ratio was equal to 1, with the rest being less than 1 (Table S6). This suggests that *GmPLATZ* probably has undergone a purifying selection pressure through the evolution of soybean.

### 2.4. Cis-Acting Element Analysis of Promoters in GmPLATZs

**Figure 5:**
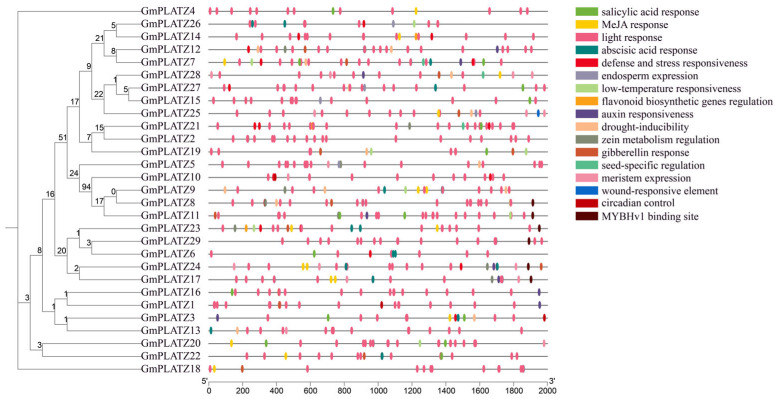
Cis-element analysis of *GmPLATZ* family gene promoters. **Left panel**: the phylogenetic relationships among *GmPLATZ* family members. **Right panel**: the promoter Cis-elements of *GmPLATZ* genes were analyzed in their 2000 bp upstream regions, with various elements high-lighted in color.

**Figure 6:**
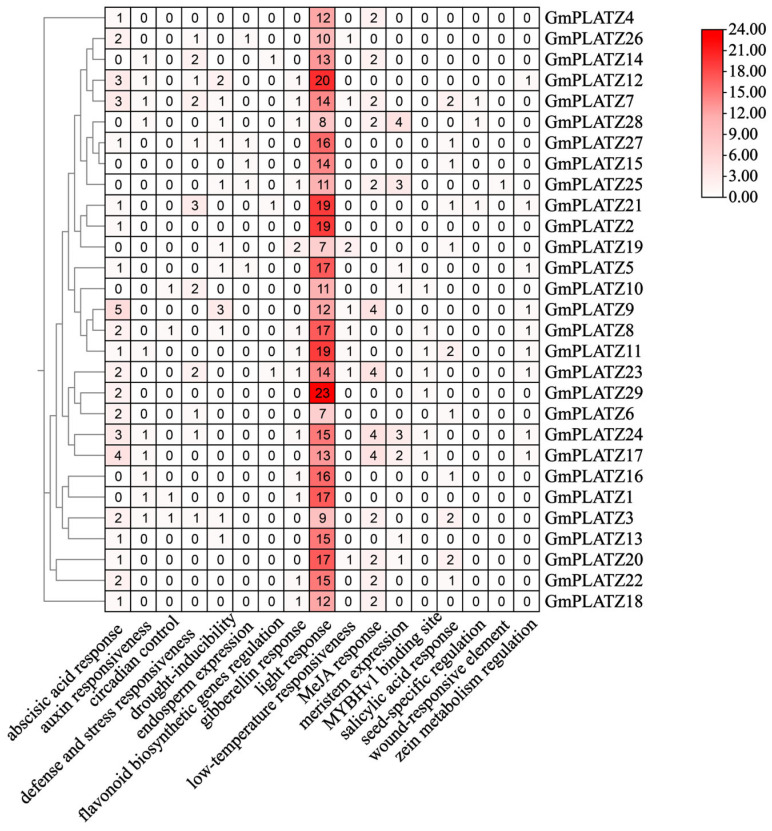
The cluster heatmap displays the number of cis-elements in different genes. Darker shades represent higher counts of specific elements in *GmPLATZs*.

In order to analyze diverse *Cis*-regulatory elements in the *GmPLATZs* promoter, we obtained the 2000 bp upstream sequences of each *GmPLATZs* gene. Subsequently, we carried out the visualization of these *Cis*-regulatory elements to analyze their characteristics. Within the *GmPLATZs* promoter regions, a total of 17 conserved elements were identified (Figure 5 and Table S7). Among the findings were responsiveness to light and hormones, stress and defense, and seed-specific regulatory elements, along with other types of elements. All *GmPLATZ* genes contain light-responsive elements, with most members additionally possessing drought-inducible and abscisic acid (ABA)-responsive elements, suggesting their potential functional roles in salt stress responses. Notably, the *GmPLATZ* family exhibits the highest abundance of light-responsive regulatory elements, along with substantial numbers of elements associated with ABA and methyl jasmonate (MeJA) responses (Figure 6). These findings suggest that the expression of *GmPLATZs* may be controlled by a variety of elements. Additionally, it is further indicated that GmPLATZs may play vital roles in soybean normal growth, development, and adaptation to environmental stresses.

### 2.5. Expression Analysis of GmPLATZs in Different Tissues of Soybean

**Figure 7:**
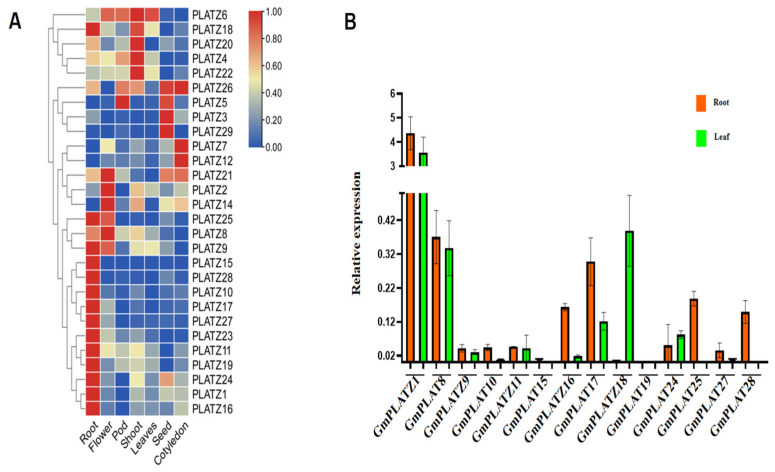
The transcriptome expression levels of the *GmPLATZ* gene family in different organs. (**A**) Heatmap of *GmPLATZ* genes expression in the root, flower, pod, shoot, leaves, seed, and cotyledon represented by a color gradient from blue to red, indicating low to high expression levels. (**B**) The expression levels of *GmPLATZ* genes between root and leaf tissues. Statistical analyses were performed using a one-way analysis of variance (ANOVA), followed by a Duncan’s multiple range test (*p* < 0.05, *n* = 3).

To investigate the tissue-specific expression of *GmPLATZ* genes in soybean, transcriptome data were collected from the Soybean Expression Atlas database (Table S8), and the expression patterns were visualized in a heatmap (Figure 7A). Among the 29 *GmPLATZ* genes, 28 were detected in the transcriptome datasets, while only *GmPLATZ13* was not detected. The results showed that *GmPLATZ6* was highly expressed in flowers, pods, shoots, and leaves. However, other individual genes exhibited a tissue-specific distinct expression pattern. For instance, among the *GmPLATZ* family genes, *GmPLATZ1*, *GmPLATZ9*, *GmPLATZ10*, *GmPLATZ11*, *GmPLATZ15*, *GmPLATZ16*, *GmPLATZ17*, *GmPLATZ18*, *GmPLATZ19*, *GmPLATZ23*, *GmPLATZ24*, *GmPLATZ25*, *GmPLATZ27*, and *GmPLATZ28* showed the highest expression in roots, while *GmPLATZ2*, *GmPLATZ8*, *GmPLATZ14*, and *GmPLATZ21* were predominantly expressed in flowers. Similarly, *GmPLATZ4*, *GmPLATZ20*, and *GmPLATZ22* exhibited peak expression in shoots, whereas *GmPLATZ3* and *GmPLATZ2* displayed extremely high expression in seeds. Additionally, *GmPLATZ7*, *GmPLATZ12*, and *GmPLATZ26* were most strongly induced in cotyledons, and *GmPLATZ5* reached its maximum expression in pods. These results suggest that the functional diversification of *GmPLATZ* genes exist in tissue-specific biological processes across the soybean.

To validate the *GmPLATZ* expression database, total RNA was extracted from both roots and leaves, followed by RT-qPCR analysis to examine the expression levels of 14 selected *GmPLATZ* genes (Figure 7B). The results showed that *GmPLATZ1*, *GmPLATZ10*, *GmPLATZ15*, *GmPLATZ16*, *GmPLATZ17*, *GmPLATZ25*, and *GmPLATZ28* were significantly upregulated in roots compared to leaves, consistent with the database. In contrast, *GmPLATZ18* exhibited the higher expression in leaves, while *GmPLATZ8*, *GmPLATZ9*, *GmPLATZ11*, *GmPLATZ19*, *GmPLATZ24*, and *GmPLATZ27* displayed similar expression levels in both tissues.

### 2.6. Expression of GmPLATZ Genes Under Salt Stress

**Figure 8:**
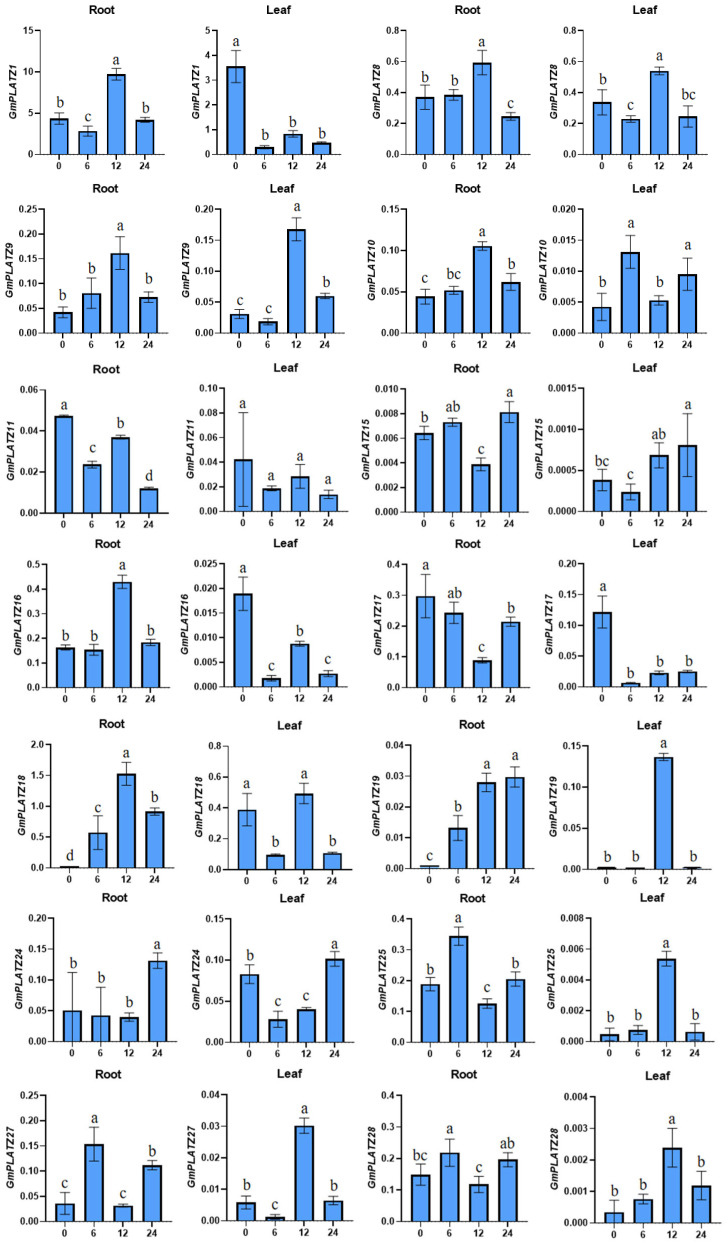
The RT-qPCR analysis was performed to examine the expression of 14 *GmPLATZ* genes in roots and leaves under salt stress. Expression data were normalized to the *Cons4* reference gene, with error bars representing standard deviations (SDs). Statistical analyses were performed using one-way analysis of variance (ANOVA), followed by the Duncan’s multiple range test (*p* < 0.05, *n* = 3). Significant differences among groups are denoted by distinct lowercase letters.

To examine the expression profiles of *GmPLATZ* genes under salt stress, soybean roots and leaves were harvested at selected time points (0 h, 6 h, 12 h, and 24 h) following salt treatment (200 mM NaCl), and the transcript levels of 14 *GmPLATZ* genes were analyzed through RT-qPCR (Figure 8). The 200 mM NaCl treatment, as a severe salt stress condition, enables faster and more intuitive observation of gene responses to salt stress [43]. The expression profiles of *GmPLATZ* genes exhibited distinct temporal and tissue-specific patterns under salt stress conditions. *GmPLATZ8* and *GmPLATZ9* demonstrated minimal basal expression in both leaves and roots at 0 h, reaching peak transcript levels at 12 h before declining to lower levels by 24 h. In contrast, *GmPLATZ19* showed differential regulation between tissues, maintaining a low initial expression in both tissues at 0 h but displaying a sustained upregulation in roots that peaked at 24 h while showing a transient leaf-specific expression detectable only at 12 h. A delayed response pattern was observed for *GmPLATZ25*, *GmPLATZ27*, and *GmPLATZ28* in leaf tissues, with maximal induction occurring at 12 h post-treatment followed by a significant downregulation at 24 h. Notably, five *GmPLATZ* family members (*GmPLATZ1*, *GmPLATZ10*, *GmPLATZ16*, *GmPLATZ18*, and *GmPLATZ24*) maintained constitutive expression throughout all root developmental stages examined, demonstrating significant upregulations either at 12 h or 24 h post-treatment. The salt stress induced divergent regulatory responses in leaf-expressed genes. Three members (*GmPLATZ1*, *GmPLATZ16*, and *GmPLATZ17*) exhibited their maximum transcript accumulation at 0 h, followed by a progressive downregulation over the treatment period. Similarly, in root tissues, *GmPLATZ11* and *GmPLATZ17* displayed analogous suppression patterns, with peak expression at initial time points preceding gradual transcriptional attenuation.

## 3. Discussion

In recent years, the rapid development of bioinformatics has provided the necessary tools for in-depth exploration of plant growth and development, as well as the identification of stress-resistance genes. Analyzing gene families represents an efficient research strategy to study the evolution, structure, and function of genes. For example, the *PLATZ* gene family has been identified in various crops and vegetables, such as tomato (*Solanum lycopersicum*) [44] and barley (*Hordeum vulgare*) [45], with studies demonstrating its association with abiotic stress responses. Previous studies have reported the phylogenetic tree analysis of the soybean *PLATZ* family. In one of these studies, the author employed the *Glycine max* Wm82.a2.v1 database and identified 31 genes belonging to the soybean *PLATZ* family [46]. In contrast, our current research, which involved analyzing the *Glycine max* Wm82.a6.v1 database, revealed 29 genes within the soybean *PLATZ* family. This difference from previous findings is likely due to the presence of pseudo genes among certain genes in the older database. However, systematic characterization of the *PLATZ* gene family in soybean (*Glycine max*) remains unreported. Through genome-wide identification, 29 *GmPLATZ* genes were systematically identified in the soybean genome. The soybean *PLATZ* family exhibits significant expansion compared to related species, containing approximately 2.6-fold more members than tomato [44] and 2.1-fold more than barley [45]. This expansion could be attributed to the larger genome size of soybean (1.1 Gb) versus tomato (900 Mb) and barley (5.1 Gb), suggesting potential whole-genome duplication events driving *GmPLATZ* diversification. Chromosomal mapping revealed uneven distribution patterns: *GmPLATZ* genes were absent from five chromosomes (Chr04, Chr14, Chr16, Chr18, and Chr20) while clustering predominantly on chromosomes 1 (4 genes) and 11 (4 genes), indicating possible evolutionary hotspots.

Genes acquire functional diversity through conserved protein domains and exon–intron architectures, which govern their molecular functions [47,48]. While exons directly encode protein sequences, introns—traditionally considered non-coding regions—undergo splicing and degradation during RNA processing. Notably, emerging studies reveal that introns serve as evolutionary drivers in plants, facilitating functional innovation through multiple mechanisms, such as alternative splicing regulation and transposable element insertion [49,50]. To investigate the evolutionary relationships of the *GmPLATZ* gene family, a comprehensive analysis of conserved domains and exon–intron architectures was conducted [51,52,53]. The analysis revealed that all *GmPLATZ* members contain three–five exons, with the majority exhibiting four exons. Most *GmPLATZ* genes harbor three introns, while a small subset possess two or five introns. Intron loss is closely associated with biological evolution [54]. Typically, intron numbers remain conserved within subfamilies, and variations across genes may arise from insertion/deletion (indel) events. Introns regulate gene expression through both positive and negative mechanisms, which may confer evolutionary advantages. Consequently, the functional diversity of *GmPLATZ* genes could be influenced by conserved and variable structural features [55].

Notably, an in-depth analysis of *GmPLATZ* proteins revealed that each member harbors a characteristic B-box zinc-binding domain, which is a hallmark feature of both the PLATZ and BBOX superfamilies (Figure 2). Phylogenetic tree construction and subsequent cladistic analysis demonstrated that the *PLATZ* family can be distinctly categorized into five well-defined subfamilies. This structural diversity strongly implies the occurrence of historical gene duplication events, or alternatively, convergent evolutionary processes that have shaped the current composition of the PLATZ gene family. Furthermore, a comparative phylogenetic examination of *GmPLATZs* and *AtPLATZs* unveiled a conserved distribution pattern within specific evolutionary clades, indicative of shared ancestral origins. Conversely, marked divergence in distribution patterns was observed across different clades (Figure 3), suggesting the influence of lineage-specific evolutionary forces on the diversification of these gene families.

The unequal distribution of genes within the *GmPLATZ* family is strongly associated with segmental duplication events in soybean. Our analysis identified 32 pairs of segmental duplication events, whereas no tandem duplication events were detected. Segmental duplication, a prevalent phenomenon in plant genome evolution, is often linked to WGD events, which are thought to drive functional diversification of gene families and enhance plant adaptability to abiotic stresses. Further collinearity analyses across *Glycine max*–*Glycine soja* and *Glycine max*–*Arabidopsis thaliana* genomes revealed striking evolutionary conservation of *GmPLATZ* genes, particularly between soybean species. This conservation likely reflects the indispensable role of *PLATZ* family genes in abiotic stress responses during evolution, prompting their retention in closely related genomes. In contrast, significant divergence and potential gene loss were observed in distantly related *Arabidopsis*, presumably due to adaptive evolution under distinct environmental pressures. Regarding the selection pressure analysis, the ratio of nonsynonymous (Ka) to synonymous (Ks) substitutions (Ka/Ks) serves as a critical evolutionary metric: values > 1 indicate positive selection driving adaptive evolution or functional innovation, while Ka/Ks < 1 signifies purifying selection favoring conservation of the protein function by eliminating deleterious nonsynonymous mutations [56]. Notably, all 32 *GmPLATZ* gene pairs in this study exhibited Ka/Ks ratios < 1, providing strong evidence for an intense purifying selection during soybean evolution. This evolutionary constraint likely facilitated the maintenance of critical stress-responsive functions, thereby enhancing soybean adaptability to diverse environmental fluctuations.

*Cis*-acting regulatory elements are essential for gene expression patterns that are specific to tissues or responsive to stress, functioning as molecular switches to fine-tune plant physiological processes during adverse conditions and thereby enhancing adaptive responses to environmental stresses [57]. Promoter sequence analysis of *GmPLATZ* genes revealed that the majority harbor *Cis*-elements associated with pathogen defense and general stress responses, strongly indicating their active involvement in abiotic and biotic stress tolerance mechanisms. Furthermore, the identification of hormone-responsive *Cis*-elements—including those for salicylic acid, abscisic acid, and jasmonic acid—within *GmPLATZ* promoter regions underscores potential integration with plant hormonal signaling networks. These findings suggest that *GmPLATZ* genes may exert their stress-mitigating functions by responding to multiple hormonal cues, enabling coordinated regulation of stress-responsive pathways of adaptation.

The association between the *PLATZ* gene family and plant salt tolerance has been sporadically reported. In Arabidopsis thaliana, *AtPLATZ2* acts as a transcriptional repressor to regulate the salt stress response by inhibiting the expression of CBL4/SOS3 and CBL10/scasp8. In cotton, *GhiPLATZ17* and *GhiPLATZ22* play critical roles in enhancing the salt tolerance of Gossypium hirsutum. However, reports in soybean remain remarkably limited [58]. In this study, RNA-seq datasets from the Soybean Expression Atlas were mined to characterize transcript abundance across diverse tissues. Comparative analysis revealed divergent tissue-specific expression patterns among *GmPLATZ* family members: some genes exhibited constitutive expression across all tissues, while others displayed tissue-restricted expression or undetectable transcription, potentially reflecting functional divergence or redundancy within the *PLATZ* gene family (Figure 7). To validate the RNA-seq results, RT-qPCR analyses were performed on 14 *GmPLATZ* genes that showed induced expression in both leaves and roots. Under salt stress conditions, distinct expression profiles were observed between leaves and roots: *GmPLATZ9*, *GmPLATZ25*, *GmPLATZ27*, and *GmPLATZ28* exhibited significant upregulation in leaves, whereas *GmPLATZ8*, *GmPLATZ10*, *GmPLATZ16*, *GmPLATZ18*, *GmPLATZ19*, and *GmPLATZ24* were markedly induced in roots. Conversely, *GmPLATZ1*, *GmPLATZ11*, *GmPLATZ16*, and *GmPLATZ17* showed significant downregulation in leaves, with *GmPLATZ11* and *GmPLATZ17* also demonstrating reduced expression in roots (Figure 8). These differential responses strongly suggest functional involvement of *GmPLATZ* genes in salt stress adaptation. Given the limited current understanding of *PLATZ* gene functions in soybean, further investigations integrating molecular biology techniques and transgenic approaches are essential to characterize their biological roles. Additionally, conducting further explorations of their regulatory mechanisms under salt stress will supply critical genetic resources and groundbreaking strategies for breeding salt-tolerant soybean varieties.

## 4. Materials and Methods

### 4.1. Authentication of Candidate GmPLATZ Genes in Soybean

The hidden Markov model of the soybean B-box zinc-binding domain was retrieved from the Pfam database (https://www.ebi.ac.uk/interpro/entry/pfam/, accessed on 25 January 2025) [59]. The TBtools software was used to screen for soybean gene proteins containing the B-box zinc-binding domain, which were subsequently named PLATZ proteins [60]. Multiple alignments of the *GmPLATZ* gene sequences and protein sequences with the *PLATZ* gene family in *Arabidopsis thaliana* were performed to further identify the GmPLATZ family. The physicochemical properties of the GmPLATZ family proteins were predicted using the ExPASy website (https://web.expasy.org/protparam/, accessed on 25 January 2025) [61], and the corresponding subcellular localization was analyzed using the Cell-PLoc 2.0 website (available at http://www.csbio.sjtu.edu.cn/bioinf/Cell-PLoc-2/, accessed on 25 January 2025) [62].

### 4.2. Characterization of Chromosomal Location, Conserved Domain, and Structure Distribution of GmPLATZs

The positions of exons and introns of *GmPLATZ* genes were identified on the Phytozome website (https://phytozome-next.jgi.doe.gov/, accessed on 25 January 2025), utilizing the *Glycine max* Wm82.a6.v1 version. Subsequently, two online resources, NCBI [63] and MEME [64], were employed to analyze the domains and conserved motifs of the GmPLATZ proteins. Finally, the chromosomal structures, locations, motifs, and conserved domains were visualized as images using the TBtools software [65].

### 4.3. Phylogenetic Classification and Analysis

The phylogenetic analysis was conducted using the PLATZ protein sequences from Arabidopsis and soybean. With the MEGA_X_10.1.7 program, multiple alignment analyses and the construction of a phylogenetic tree were carried out. This process adopted the default parameters, specifically applying the neighbor-joining method and incorporating 1000 bootstrap iterations.

### 4.4. GmPLATZ Gene Synteny Analyses and Duplication

Tandem and segmental duplication events of *GmPLATZ* in the soybean genome were analyzed using TBtools with the GFF (General Feature Format) file. The Dual Synteny Plotter module was then employed to examine the homology between *Arabidopsis thaliana* and soybean, as well as between Glycine soja and soybean. Additionally, the non-synonymous substitution (Ka), synonymous substitution (Ks), and Ka/Ks ratio were calculated [65].

### 4.5. Promoter Cis-Regulatory Elements Analysis of GmPLATZs

The 2000 bp upstream sequences of the *GmPLATZ* genes were extracted from the Phytozome (https://phytozome-next.jgi.doe.gov/, accessed on 25 January 2025) website. Corresponding cis-acting elements were then identified using the PlantCARE database (http://bioinformatics.psb.ugent.be/webtools/plantcare/html/, accessed on 25 January 2025) [66], followed by visualization of the cis-regulatory elements in *GmPLATZ* promoters through TBtools software [65].

### 4.6. Expression Pattern Analysis of GmPLATZ Gene Family in Tissues

To explore the expression patterns of *GmPLATZ* genes, transcriptional levels of *GmPLATZ* gene members across different tissues were obtained, which was achieved by querying the Soybean Expression Atlas (https://soyatlas.venanciogroup.uenf.br, accessed on 7 January 2025). The Atlas offers high-resolution gene expression data from a wide range of fourteen tissues, including cotyledon, shoot, leaf, root, flower, seed, and pod, during the whole developmental stage of soybean [67]. Subsequently, the TBtools software [65] was utilized to generate a tissue expression heatmap for visualization.

The response of *GmPLATZs* to external stimuli was investigated using online transcriptome data. Specifically, the study analyzed *GmPLATZ* gene expression patterns in roots and leaves of Williams 82 soybean seedlings.

### 4.7. RNA Extraction and Reverse-Transcription Quantitative PCR (RT-qPCR) Analysis

Soybean cultivar Williams 82 seedlings were subjected to two treatments: normal water and 200 mM NaCl. The Williams 82 seeds were provided by the Hybrid Soybean Team of Jilin Academy of Agricultural Sciences. The seeds were first rinsed with tap water to remove surface debris, followed by sterilization via immersion in a 0.1% sodium hypochlorite solution for 5 min. After three rinses with distilled water, the seeds were sown in vermiculite and placed in a Conviron incubator set at 25 °C with 60% humidity under a 16 h light/8 h dark photoperiod. Upon reaching the V1 growth stage, the plants were subjected to salt solution treatment. Samples were collected at 0, 6, and 12 h after treatment. For both the control (normal water) and treatment (200 mM NaCl) groups, roots and leaves were harvested, with three biological replicates each. Immediately after collection, the samples were rapidly frozen in liquid nitrogen to preserve their integrity, which is essential for total RNA isolation. The RNA extraction process adhered strictly to the guidelines provided with the EasyPure Plant RNA Kit (Cwbio, Beijing, China). After obtaining the RNA, 1 µg of it was used for reverse transcription. This step was carried out using a TransScript All-in-One First-Strand cDNA Synthesis SuperMix for qPCR (Trans, Beijing, China), enabling the conversion of RNA into complementary DNA suitable for subsequent quantitative real-time PCR analysis. The expression levels of *GmPLATZ1*, *GmPLATZ8*, *GmPLATZ9*, *GmPLATZ10*, *GmPLATZ11*, *GmPLATZ15*, *GmPLATZ16*, *GmPLATZ17*, *GmPLATZ18*, *GmPLATZ19*, *GmPLATZ24*, *GmPLATZ25*, *GmPLATZ27*, and *GmPLATZ28* were detected via the RT-qPCR. The RT-qPCR was performed using 2 × SYBR Green qPCR Mix kit (Trans, Beijing, China) on the applied biological systems by Thermo Fisher Scientific (Quant Studio Laboratories, Hercules, CA, USA). For SYBR Green-based amplification, Perfect Start Green qPCR Super Mix was utilized. The 10-μL reaction system consisted of 5 μL of Perfect Start Green qPCR Super Mix, 0.2 μL of forward primer, 0.2 μL of reverse primer, 100 ng of cDNA, and nuclease-free water to adjust the volume to 10 μL. The two-step qRT-PCR conditions were as follows: pre-denaturation: 95 °C for 30 s (1 cycle); denaturation and annealing–extension: 95 °C for 15 s, 60 °C for 1 min (40 cycles); melting-curve analysis: 95 °C for 15 s, followed by 60 °C for 1 min and 95 °C for 1 s (1 cycle each). *Cons4* (BU578186) was used as the reference gene, and its specific primers are listed in Table S9. The relative expression levels were calculated using the method established by Livak and Schmittgen.

### 4.8. Statitical Analysis

In this study, a Student’s *t*-test in Graphpad Prism 8 (Version 8.0, Motulsky, San Diego, CA, USA) was used to analyze the differences between the different treatments. A *p*-value cut-off of 0.05 was considered as statistical significance. All the error bars were standard errors (SEs).

## 5. Conclusions

In this study, we systematically identified 29 *GmPLATZ* genes in the soybean genome and conducted comprehensive characterization of this family’s genes. Integrated analyses of gene structure, protein physicochemical properties, and conserved domains revealed that members within the same subfamily exhibited similar structural features. Phylogenetic and synteny analyses demonstrated that segmental duplication played a major role in the expansion of *GmPLATZ* genes during soybean genome evolution. Most *GmPLATZ* genes within the same subfamily contained identical cis-regulatory elements and conserved motifs in their promoter regions, particularly those associated with salt stress responses. However, certain discrepancies were observed, suggesting potential functional diversification among *GmPLATZ* members. Tissue-specific expression patterns indicated that these genes might play distinct regulatory roles in soybean growth and development. Notably, 14 *GmPLATZ* genes showed differential expression patterns in response to salt stress, and among the 14 *GmPLATZ* genes, the *GmPLATZ18* and *GmPLATZ19* genes are significantly regulated by salt stress in soybean roots, implying their potential key roles in salt tolerance. Further in-depth functional exploration of these two genes could be conducted in the next step. Functional characterization of *GmPLATZ* genes could significantly contribute to improving the soybean yield and stress resistance, offering potential targets for molecular breeding strategies.

## Data Availability

All data are contained within the article.

## Supplementary Materials

The following supporting information can be downloaded at https://www.mdpi.com/article/10.3390/plants14132004/s1: Table S1: List of the identified *GmPLATZ* genes and their related information in soybean; Table S2: Analyses the motifs in GmPLATZ from the MEME website; Table S3: *AtPLATZ* genes in *Arabidopsis thaliana*; Table S4: Tandemly and segmentally duplicated *GmPLATZ* gene pairs; Table S5: One-to-one orthologous relationships between the *PLATZ* gene members in Glycine max and *Arabidopsis thaliana*; Table S6: One-to-one orthologous relationships between the *PLATZ* gene members in Glycine max and Glycine soja; Table S7: Cis-element analyses of the *GmPLATZ* gene promoter regions; Table S8: Expression profiles of *GmPLATZ* genes in multiple tissues throughout various developmental stages; Table S9: Primers used in this study for RT-qPCR; Figure S1: Amino acid sequence alignment of the 29 GmPLATZ proteins; Figure S2: The treatment with 200 mM NaCl solution for Williams 82.

## Abbreviations

The following abbreviations are used in this manuscript:

NF-Y	Nuclear Factor Y
MYB	Myeloblastosis
GRF	Growth-Regulating Factor
AP2	APETALA2
