# Supplementary material for: Genome-Wide Identification and Salt Stress-Responsive Expression Analysis of the GmPLATZ Gene Family in Soybean (Glycine max L.)
Source: Plants (Basel). 2025 Jun 30;14(13):2004. doi: 10.3390/plants14132004 (PMC12251706; doi:10.3390/plants14132004)
Supplement: Supplementary file 1 [file plants-14-02004-s001.zip › supplementary.pdf]

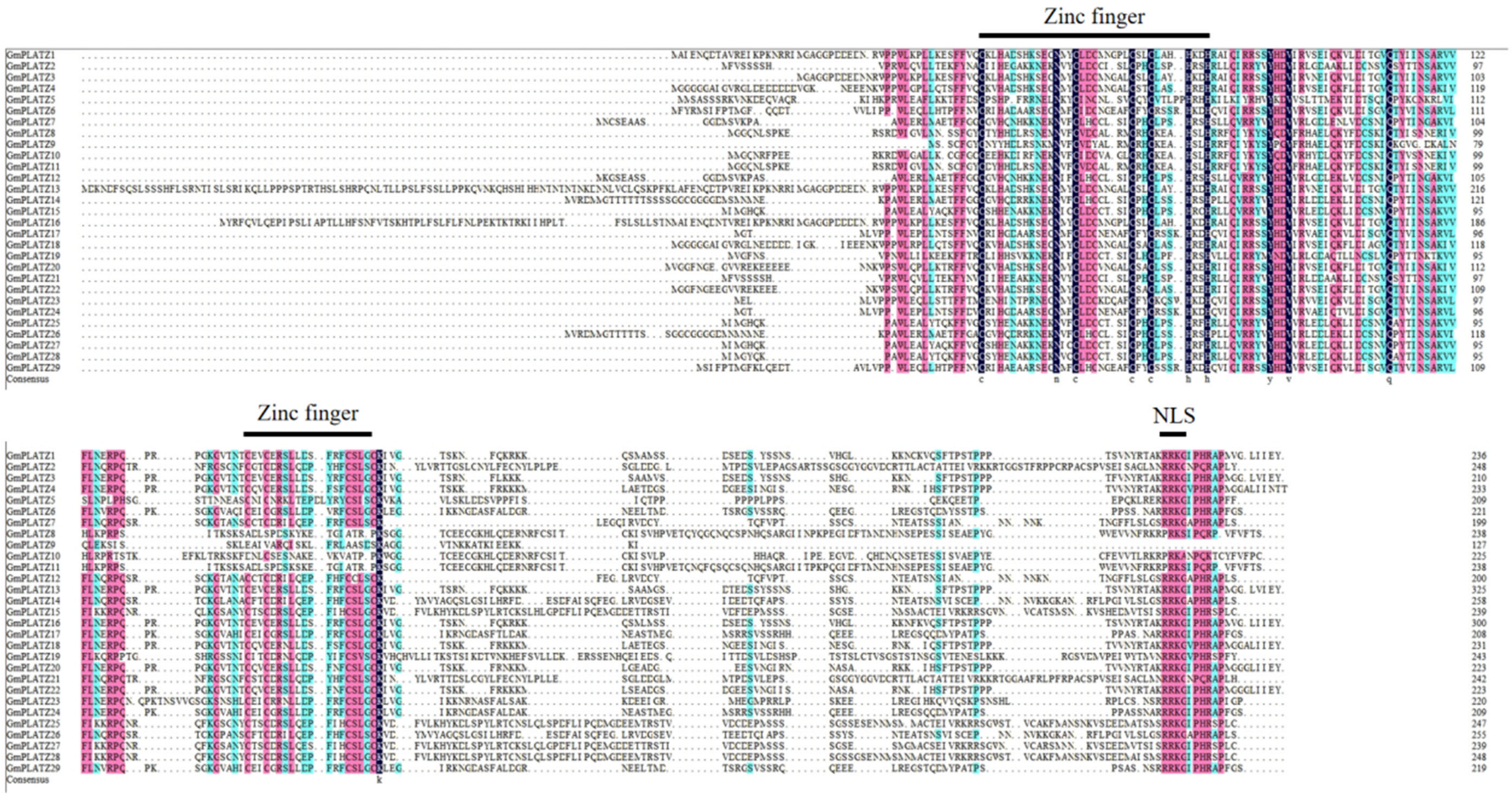

Figure S1 Amino acid sequence alignment of the 29 GmPLATZ proteins.

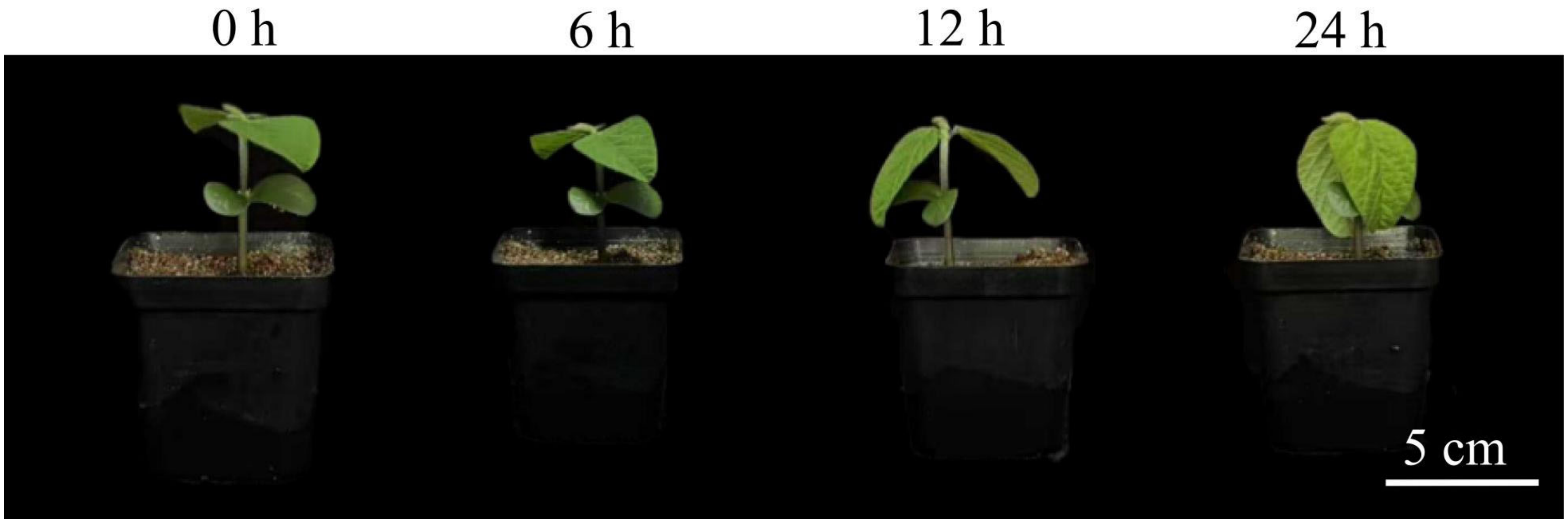

Figure S2 The treatment with 200 mM NaCl solution for Williams 82.
